# Multidisciplinary Allied Health Reablement Model of Care for Older People in Residential Aged Care and Community Settings: Mixed-Methods Evaluation

**DOI:** 10.1177/07334648251351690

**Published:** 2025-06-24

**Authors:** Laura Mo, Katharine Scrivener, Alice Pashley, Diane Gibson, Kasia Bail, Nathan D’Cunha, Stephen Isbel

**Affiliations:** 1 Concentric Healthcare Services, Sydney, NSW, Australia; 2 7788 Macquarie University, Sydney, NSW, Australia; 3 2234 University of Canberra, Canberra, ACT, Australia

**Keywords:** nursing home, home care services, allied health occupations, multidisciplinary, reablement

## Abstract

This study aimed to explore the feasibility, acceptability, and clinical outcomes of a best practice–aligned multidisciplinary allied health reablement model of care for older people. A mixed-methods pre-post-intervention study was conducted in two nursing homes and the community. Quantitative measures were collected for frailty, physical function, and quality of life for all participants pre-implementation and 12 weeks post-implementation. Semi-structured interviews were conducted with a sub-group of participants and allied health professionals involved in the intervention. Participants’ (*n* = 50) physical function increased (SPPB 4.2 vs. 4.9) while frailty (FRAIL-NH 6.0 vs. 5.5) and quality of life (16 vs. 16) were maintained. There was a high retention (93%) and attendance rate (84%), indicating acceptability. Participants received a daily median of 16 allied health minutes, costing $26AUD. Findings confirm acceptability and feasibility of the model with potential to maintain or improve clinical outcomes. Future work is needed to define long-term outcomes, scalability and sustainability. The study was registered with the ANZCTR [Trial ID: ACTRN12623000915651; Registration Date: 12/1/2024].

**What this paper adds**

• A reablement-focused, multidisciplinary allied health model of care (REABLE-MOC) for older people that services both residential aged care and community settings is feasible and acceptable.
• The REABLE-MOC supports job satisfaction and growth, addressing key barriers to workforce issues in aged care.
• Best practice allied health services for aged care can maintain or improve functional and quality of life outcomes.

**Applications of Study Findings**

• Aged care services should enable allied health professionals to work at a greater scope-of-practice.
• Future research should also explore aged care staff knowledge, skills, and practices regarding reablement for older people.

## Introduction

Australia’s ageing population is projected to increase due to greater longevity and population growth. Currently, 1.2 million people are aged 80 and over, a figure expected to double within 20 years (Australian Bureau of Statistics, 2023). In addition, demand for aged care services that promote autonomy, quality of life, and ageing in place at home is increasing (Australian Institute of Health and Welfare [AIHW], 2024). Reablement has emerged as a key approach that addresses declines in functional independence and well-being against the background of broader reforms emphasizing individualized, rights-based care (Department of Health and Aged Care, 2023b). Reablement is a goal-orientated, person-centered approach delivered by a coordinated team to enhance or maintain an individual’s physical and overall functioning (Metzelthin et al., 2022). In contrast, the current Allied Health Professional (AHP) service delivery in Residential Aged Care (RAC) has been described as a reactive approach to referrals for individual disciplines (Allied Health Professionals Australia, 2022), which doesn’t support reablement approaches. AHPs play an integral role in effective reablement (Buma et al., 2022), with positive impacts of allied health interventions in aged care observed (Bennett et al., 2022; Meulenbroeks et al., 2022; Mitterfellner et al., 2024). The revised Aged Care Quality Standards recognize AHPs’ role in comprehensive delivery of aged care services to maintain older people’s functional capabilities and well-being (Department of Health and Aged Care, 2023b).

Since 2022, there has been a decline in the median time people in RAC have received allied health services and related spending (Gibson & Isbel, 2024). Australians living in RAC averaged receiving 4.2 minutes per day of allied health services in 2024 (Department of Health and Aged Care, 2024c), reduced from 5.6 minutes reported in 2022 (Department of Health and Aged Care, 2023a). This decline persists despite the Royal Commission into Aged Care Quality and Safety (2021) recommending improvements in allied healthcare (Australian Government, 2021), with 22 minutes per day as the international best practice standard (Eagar et al., 2019). The disconnect between recommendations and actual practices likely arose from employment models that do not support quality allied health services, for example, ad hoc or contract work (Bartrim et al., 2024; Luongo et al., 2022), combined with mandatory requirements to increase nursing and personal care staffing levels, with associated increases in expenditure (Gibson & Isbel, 2024). Similarly with community-based aged care, the Commonwealth Government requires principles of “wellness and reablement” to be embedded in service models; however, significant challenges have hindered implementation of these approaches (Department of Health and Aged Care, 2024b). Challenges include service access delays, insufficient funding, and workforce constraints resulting in reduced delivery of reablement-focused allied health services (Department of Health and Aged Care, 2024a). These systemic constraints in both RAC and community settings are a significant barrier to older Australians being able to live independently and engage in meaningful activities.

There is a need to implement and evaluate multidisciplinary care models that effectively deliver reablement services aligned with best practice. Higher levels of allied health provision are associated with reduced falls, improved care quality, and participation in activities of daily living (Meulenbroeks et al., 2022). Employment models embedding AHPs within RAC may facilitate higher levels of AHP provision, enabling increased access and greater quality of care. Allowing community members to engage these services may also support access to reablement in the community, increasing the reach of these approaches for older Australians. This study aimed to explore the feasibility, acceptability, and clinical outcomes from delivering a reablement-focused, multidisciplinary allied health model of care (REABLE-MOC) for older people in RAC and the community aligned with best practice.

## Methods

### Design

A pre-post-trial of the REABLE-MOC was conducted. A convergent mixed-methods approach was taken, where quantitative and qualitative data were collected in parallel and integrated during analysis (Fetters et al., 2013) to support a comprehensive understanding of feasibility, acceptability, and outcomes of the model. This study was situated in a pragmatic paradigm, which “orients itself toward solving practical problems in the ‘real world’” to support the study aims (Yvonne Feilzer, 2010, p. 8). The study was registered with the ANZCTR (Trial ID: ACTRN12623000915651; Registration Date: 12/1/2024). Ethics approval was obtained from University of Canberra Human Research Ethics Committee (HREC-3306).

### Setting

The study was conducted in two RAC homes in Sydney, Australia, and the immediate community within a 5-kilometer radius of the RAC homes. The RAC homes were large (more than 100 beds) and provided 24-hour care for residents who required assistance in daily activities, mobility, and complex healthcare management.

### Model of Care

The REABLE-MOC was developed in consultation with AHPs, nurses, general practitioners, and clinical service leaders experienced in working with older people, and informed by a related project (Isbel et al., 2025). The model draft was then reviewed and finalized with the research team.

**Figure 1.**
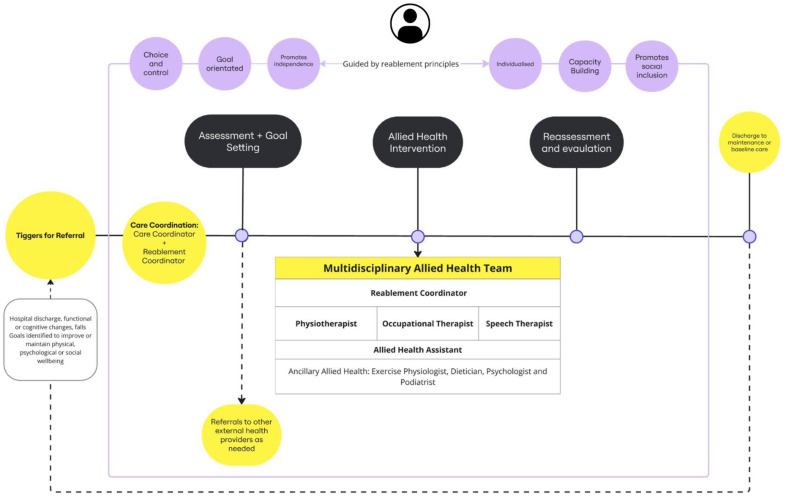
Reablement-focused, multidisciplinary allied health model of care.

The REABLE-MOC involved structured delivery of multidisciplinary reablement services delivered by a physiotherapist (PT), exercise physiologist (EP), occupational therapist (OT), speech therapist (ST), and an allied health assistant (AHA). The REABLE-MOC also included a reablement coordinator (a registered PT), who was responsible for participant screening for referrals, goal identification, appointment scheduling, leading case management meetings, and acted as the main point of contact for staff and clients. The REABLE-MOC was provided to participants for a trial period of 12 weeks. Existing staff at the services were trained to provide reablement processes according to the intervention plan. Figure 1 provides an overview of the REABLE-MOC. Key differences between traditional RAC allied health services and the REABLE-MOC include triggers for referral (traditional referrals primarily focused on issues related to risk and compliance, e.g., mobility, falls, and pain), structured goal setting, scope of practice, and intervention modality (see Table 1). Usual AHP service provision for community members was dependent on type of funding pathway available to the individual (e.g., private health, GP referral, and home care package).

**Table 1.** Summary of Allied Health Interventions.

Types of intervention and supporting evidence	Modality	Delivered by
***Cognition:*** Cognitive stimulation therapy to improve cognition and social engagement (Woods et al., 2023).	Direct intervention	Occupational therapist/speech therapist
	Group or individual program	
	**Group program:** 2 × 60 min classes per week for 8 weeks	
	Group size: 4–6 people per class	
	**Individual program:** 2 × 60 minutes per week for 8 weeks	
***Exercise:*** Multicomponent exercise program targeting strength and balance based on progressive strengthening principles and balance retraining (Hewitt et al., 2018). Exercises were specific to goals identified and adapted to participants’ level of disability.	Direct intervention	Physiotherapist/exercise physiologist
	Group or individual program	
	**Group program:** 2 × 60 min classes per week for 8 weeks	
	Group size: 6 people per class	
	**Individual program:** 2 × 30 minutes per week for 8 weeks	
***Communication:*** Direct interventions included creation and training in the use of low-tech Augmentative and Alternative Communication (AAC) to facilitate conversation or alternative communication methods such as memory books and graphic cues.	Direct and indirect intervention	Speech therapist/occupational therapist
	Group or individual program	
	**Group Interventions:** 2 × 60 min classes per week for 6 weeks	
Communication partner training to facilitate communication skills training for family members or care workers (O'Connor et al., 2019).	Group size: 4–6 people per class	
Indirect interventions included research, liaison, and resource development specific to goals identified.	**Individual intervention:** 2 × 45–60 minutes per week for 6 weeks	
***Daily activities:*** interventions based upon areas of self-care, leisure activities, and productivity (Mitterfellner et al., 2024; O'Connor et al., 2019). This included task retraining or environmental modification, use of adaptive equipment to overcome challenging everyday tasks or functional activities, engagement in valued leisure activities and productivity level goals such as volunteering or making items for donation (for example). Care worker training was provided to support daily activities.	Direct and indirect intervention	Occupational therapist
	Group or individual program	
	**Group program:** 2 × 60–90 min classes per week for 6–12 weeks	
	Group size: 4–6 people per class	
	**Individual program:**	
	2 × 45–60 minutes per week for 6–12 weeks	

### Participant Recruitment

All residents in the two RAC homes were screened for eligibility to receive the REABLE-MOC, with the assistance of the RAC clinical team. Participants in the community were recruited through flyers distributed to independent living units and local community programs. Participants were included if they were permanent residents at the included RAC homes or lived within a 5 km radius of RAC homes. Participants were excluded if they were currently accessing or had accessed allied health intervention in the previous three months or were medically advised against participation. If interested, the residents and their next of kin were approached to discuss the project, and the participant information form was shared. Written informed consent was obtained from all participants or their enduring power of attorney.

Interviews were conducted with a sub-group of participants, as well as with the AHPs involved in delivering the REABLE-MOC. Information power was used to determine a sample size of 20–25 participants would be needed for data adequacy (Malterud et al., 2016). Participants were eligible to participate in an interview if they had completed the 12-week trial of the REABLE-MOC. Participants were purposively sampled to obtain diversity in physical and cognitive function. Next of kin were invited to support participants with cognitive or communication impairments answering questions. A convenience sample of all AHP who delivered the REABLE-MOC were invited to participate in an interview. All participants provided written and verbal consent prior to interviews. Recruitment occurred between December 2023 and June 2024.

### Data Collection

Participants’ age, sex, falls history, use of mobility aids, Australian National Aged Care Classification (AN-ACC), previous health conditions, and living arrangements were extracted from the clinical management platform for participants living in RAC homes or through health summaries from the general practitioner for participants living in the community. The AN-ACC funding model assigns residents to a class from 1 to 13, which decides the funding the aged care home receives to support residents (Eagar et al., 2020). The Montreal Cognitive Assessment was completed by a trained occupational therapist to describe cognitive status at baseline (Nasreddine et al., 2005).

Clinical outcomes were measured within three days of consent and following 12 weeks of receiving the REABLE-MOC. An overview of clinical outcome measures used can be found in Table 2. Feasibility was explored using AHP employment and time for direct and indirect activities, intervention delivery and modality, session content, cost, and qualitative data. Acceptability was explored through attendance and retention rates, adverse events, and qualitative data. Feasibility and acceptability measures were recorded by the AHP following each session in an excel spreadsheet. Retention was measured as the proportion of participants completing the 12-week trial. Attendance of sessions was recorded noting rescheduled or cancelled sessions. Pre-defined descriptive codes for intervention types were used to summarize session content, including direct activities (assessments and interventions) and indirect activities (case management, training, travel, and administrative tasks). AHPs recorded codes, session time, and adverse events in clinical documentation. Program cost was calculated by multiplying total AHP and assistant time by $95/hour, reflecting study-specific conditions including location, economic context, and the skills of the team.

**Table 2.** Quantitative Measures.

Outcome	Measure	Description
Frailty	FRAIL-Nursing Home (FRAIL-NH) (Kaehr et al., 2016)	Scores ranging from 0 to 13, where 0–5 indicates non-frailty, 6–7 pre-frailty, and 8≤ frailty
Physical functioning	Short Physical Performance Battery (SPPB) (Guralnik et al., 1994)	Measure of functional capability through balance, walking, and sit-to-stand timed tasks, scored 0 (worst) to 12 (best)
Quality of life	Quality of life-Aged Care Consumers (QoL-ACC) (Cleland et al., 2021; Khadka et al., 2022)	Assesses six dimensions of mobility, pain management, emotional well-being, independence, social connection, and activities, with higher scores indicating greater quality of life.
Occupational performance	Canadian Occupational Performance Measure (COPM) (Carswell et al., 2004)	Measures self-rated satisfaction and performance scores for five participant-identified occupational performance problems. Performance and satisfaction with these each scored between 1 (worse) and 10 (best), and were averaged across the occupations, providing an overall score of 1–10.
Social engagement	Revised Index for Social Engagement (RISE) (Gerritsen et al., 2008)	Measures social interaction and participation in structured and social activities in RAC, scoring 0–6, with higher scores indicating greater engagement

*Note.* Proxy responses for the COPM, QoL-ACC, and RISE were provided when participants were unable to or declined to complete them, by next of kin or carers who provided consistent care for at least six months.

Semi-structured interviews were conducted to explore perspectives and experiences of the participants receiving the REABLE-MOC and staff who delivered the REABLE-MOC. Interviews were conducted in person or via teleconference by the lead author (LM), depending on preference. The interviewer had a working relationship with staff participants, but not the resident or next of kin participants. An interview guide (supplemental file), piloted by the research team, was shared with interviewees beforehand. The interview guides were developed to obtain feedback on experiences and processes of the program, with resident interview guides being more directive to support participants with cognitive impairment. Interviews ran for an average of 15.39 minutes (range 6.22–37.09 minutes), were audio-recorded, transcribed verbatim, and reviewed for accuracy. Transcripts were shared with participants, and field notes were completed post-interview.

### Data Analysis

Descriptive statistics were performed for quantitative data, with continuous variables reported as mean and standard deviation or median and interquartile ranges and categorical data reported as counts and proportions. Median hours, cost of delivering the REABLE-MOC for both daily (over seven days), and complete trial duration were calculated using total AHP time. The study was not powered to perform inferential statistics, but changes in pre- and post-performance were described by the mean magnitude of change from the sample and then compared with clinically meaningful differences where known.

Qualitative content analysis was performed on qualitative data following the framework method, chosen for its suitability in generating a descriptive summary and addressing questions related to acceptability and satisfaction of the model’s implementation (Gale et al., 2013). Participant and staff interviews were analyzed separately. First, data familiarization and independent coding were completed inductively by research team members (LM and SI) for a sample of interviews to create a working analytic framework. The remaining interview data were then charted against this framework, with the analytic framework iteratively developed as insights and interpretations developed. Regular meetings were held to discuss alternative interpretations to deepen insights until no new themes were developed. Data relating to clinical outcomes, and feasibility and acceptability outcomes were grouped and integrated separately. Integration of data involved comparison of the quantitative clinical outcomes against the themes produced from participant and staff qualitative analysis to determine if they were confirming, expanding, or disagreeing, and were reported narratively. A checklist for mixed-methods research (Lee et al., 2022) was used to prepare this manuscript (supplemental file).

## Results

**Figure 2.**
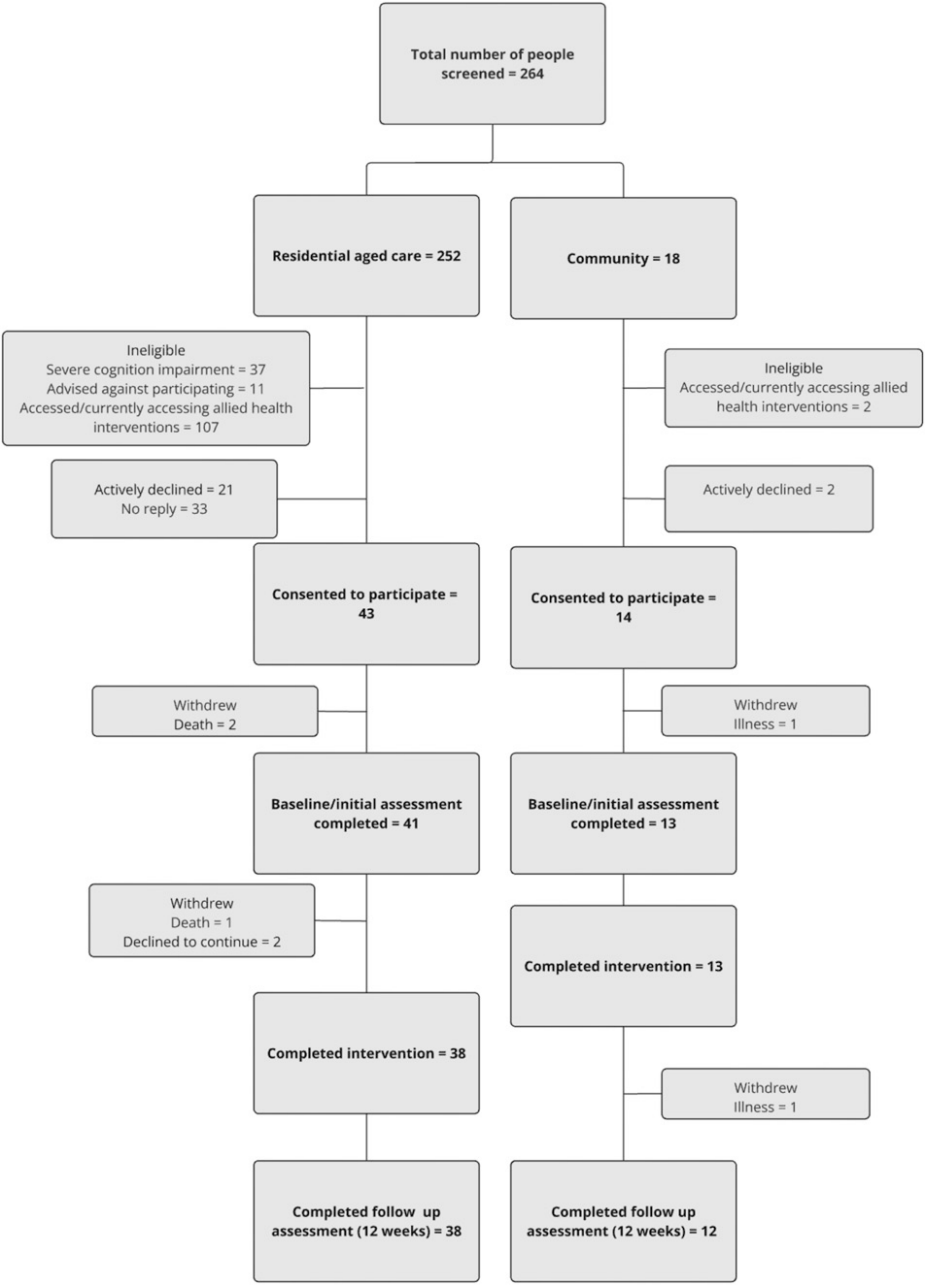
Recruitment flowchart.

A total of 252 permanent residents across RAC sites and 18 people living in the community were screened for eligibility. Fifty-seven (21%) participants were recruited to the trial, with 43 coming from RAC and 14 from community settings. Fifty participants had data available for both pre and post measures, *n* = 38 from RAC and *n* = 12 from community (Figure 2).

Participants had a mean age of 85 years (SD 6), 70% were female, and 70% used a mobility aid. The median MoCA was 13/30 (IQR 7–18) and dementia diagnosis was present in 43% of the participants. See Table 3. Interviews were conducted with *n* = 29 participants: *n* = 16 older people (*n* = 11 from RAC homes and *n* = 5 from community), six family members (supporting (*n* = 2) or representing participants (*n* = 4)), and seven AHPs (two OTs, four PTs, and one ST). Table 4 provides the themes and sub-themes from the participant and AHP interview analysis, and a detailed coding tree with exemplar quotes can be found in the supplemental file.

**Table 3.** Baseline Characteristics of Participants.

Characteristics	All (*n* = 50)	Residential aged care home (*n* = 38)	Community (*n* = 12)
Age, mean (SD)	85 (6)	85 (6)	84 (7)
Female, *n* (%)	37 (74)	31 (76)	7 (54)
Fell in prior 12 months, *n* (%)	25 (50)	20 (53)	5 (42)
Uses mobility aid, *n* (%)	35 (70)	31 (82)	4 (33)
Cognition (MoCA^a^), median (IQR)	13 (7–18)	10 (7–16)	18 (12–26)
AN-ACC,^b^ median (IQR)	N/A	8 (5–11)	N/A
Lives alone, *n* (%)	N/A	N/A	7 (54)
Health conditions, *n* (%)			
Anxiety	26 (52)	20 (53)	6 (50)
Depression	24 (48)	19 (50)	5 (42)
Cardiac disease	17 (34)	12 (32)	5 (42)
Cerebrovascular disease/stroke	12 (24)	9 (24)	3 (25)
Hypertension	32 (64)	23 (61)	9 (75)
Incontinence	34 (68)	32 (84)	2 (17)
Parkinson’s disease	4 (8)	3 (8)	1 (8)
Dementia	20 (40)	18 (47)	2 (17)

^a^MoCA = Montreal Cognitive Assessment.

^b^AN-ACC = Australian National Aged Care Classification.

**Table 4.** Overview of Themes and Sub-Themes for Participant and Allied Health Professional Qualitative Analysis.

Participant results		Allied health professional results	
Themes	Sub-themes	Themes	Sub-themes
Impact and benefits of person-centered interventions [*PCI*]	Person-centered nature of the program	Better than usual care and past practices [*Better practices*]	Goal-driven and person-centered approach
	Occupation-based interventions		Comparison to past practices and usual care
	Feelings of empowerment and purpose		Aspects of program that contributed to improved practice
	Physical, psychological, and social benefits	Importance of multidisciplinary approach [*MDT approach*]	Benefits of multidisciplinary approach for AHPs
	Retained social connects to family, social network, and community		Benefits of multidisciplinary approach for participants
Diverse role of allied health professionals [*Role of AHP*]	AHP’s role as a coach and educator	Personal and professional satisfaction [*Satisfaction*]	Satisfaction working in aged care
	Diverse range of skills and attributes were valued by participants		AHPs were able to deliver interventions to their full scope of practice
Challenges [*Challenges*]	Model sustainability	Challenges in delivering reablement [*Challenges*]	Challenges the person presented
	Limited goal achievement led to dissatisfaction		Challenges the RAC setting presented
			Improvements to program

### Clinical Outcomes and Experience

Following the intervention, *n* = 15 (39%) of RAC participants had improvements in frailty, *n* = 13 (34%) had maintained, and *n* = 10 (26%) had deteriorations. Of the community participants, three (25%) had improvements in frailty, eight (67%) maintained, and one (8%) had deteriorated. Overall, participants had a small improvement in frailty of −0.5 points (see Table 5), which is not considered clinically meaningful (Liau et al., 2021). SPPB scores showed a small mean improvement of 0.7 (SD 3.9) following completion of the trial, which is considered clinically meaningful (Perera et al., 2006). Participants reported the impact of these improvements in physical functioning [*PCI*], with one participant stating, “I can tell that I’m stronger, so I’m getting around better, shopping, everything” (P9, community).

**Table 5.** Clinical Outcomes.

	Total (*n* = 50)		RAC (*n* = 38)		Community (*n* = 12)	
	Pre	Post	Pre	Post	Pre	Post
FRAIL-NH (median, IQR)	6 (2.0–8.0)	5.5 (2.0–8.3)	7.5 (3.0–10.0)	8.0 (3.0–10.0)	2.0 (2.0–4.5)	2.0 (1.3–4.8)
SPPB (mean, SD)	4.2 (3.8)	4.9 (3.9)	3.4 (3.7)	4.1 (3.9)	6.9 (2.8)	7.4 (2.8)
Gait speed (mean, SD)	0.4 (0.4)	0.5 (0.4)	0.4 (0.4)	0.4 (0.1)	0.6 (0.03)	0.7 (0.1)
QoL-ACC^a^ (median, IQR)	16 (13.0–17.3)	16 (14.8–19.0)	16 (12.0–18.0)	16 (13.8–19.0)	15.5 (13.3–17.0)	17.0 (15.3–20.8)
COPM^a^						
Performance (median, IQR)	3.8 (2.7–5.1)	6.9 (5.6–7.8)	3.7 (2.7–5.0)	7.0 (5.7–7.7)	4.9 (2.5–6.2)	5.7 (5.1–7.2)
Satisfaction (median, IQR)	3.9 (2.3–4.8)	7.1 (5.5–8.0)	3.5 (2.3–4.5)	7.6 (5.9–8.4)	4.2 (2.3–5.4)	5.8 (5.1–7.2)
Rise^a^ (median, IQR)^b^	4.0 (3.0–5.0)	5.0 (2.0–6.0)	4.0 (2.5–5.5)	4.0 (1.5–6.0)	4.5 (4.0–4.5)	6.0 (6.0–6.0)

^a^Proxy responses included (next of kin or usual assistant in nursing/care worker).

^b^RISE scores only available for *n* = 11 participants; *n* = 9 from RAC and *n* = 2 from community.

Interestingly, participants more often described improvements to their quality of life [*PCI*], particularly around finding purpose through attending therapy.

It gave her something to live for. It gave her something to do, even though it was only for a short period… she was happy with herself, she was proud of herself…she would say, “See, I can do it.” (P14 next of kin, RAC)

However, this was not well reflected in QoL-ACC scores, which were maintained for RAC participants and were improved slightly for community participants (Table 5). Participants, carers, and AHPs often spoke of the social engagement with other participants during the therapy sessions as a valued aspect of the REABLE-MOC [*PCI; Better practices*], which was stated to have previously been “a bit overlooked” (PT2). This was confirmed through a small increase in RISE median scores (Table 5). Some participants described improvements to “my confidence, my mental state” (P15, RAC), which was attributed to the frequent and ongoing contact between participants and the AHP [*Role of AHP*], who provided encouragement and tailored advice.

But then when you go into the group, you find that other people have the same problems as you have, and you can talk together with those people, and together with your therapist find ways of overcoming any problems that you might have. (P7, RAC)

Occupational performance and satisfaction demonstrated improvements of 2.6 (IQR1.2–3.6) and 3.2 (IQR1.5–4.5) points, respectively (Table 5), representing clinically meaningful changes (Carswell et al., 2004). The personalized approach to improving occupational performance was highly valued by participants for enabling control, autonomy, and feelings of worthiness [*PCI*].

I was asked what I would like to do, and that made me feel a little bit more in control of my life… being able to say how you'd like to do something makes you feel, again, that you are worthwhile. (P7, RAC)

Participants and their carers reported the benefits of the model on activities of daily living, which had supported regaining independence in activities such as feeding or community mobility. However, not all participants saw improvements in their occupations, with some noting dissatisfaction with the lack of progress [*Challenges*] (“My mobility has not improved. It’s worsened, I think, and that’s upsetting to me,” P2, RAC). Participants appreciated exploration of hobbies that they had not been able to engage in since entering RAC, particularly when modifications were provided to enable them to re-engage with their friends and communities.

We just mentioned scrap booking in passing, and now we've done it. I'm thoroughly enjoying it… I mean, you just got more involved in it, the more videos you saw about it… That’s something I will keep doing, as well. It helps pass the time of day. (P1, RAC)

### Acceptability and Feasibility

The REABLE-MOC was delivered with three OTs (0.8FTE), four PTs (1.0FTE), and one ST (0.1FTE) for RAC and community participants. The AHA provided interventions for RAC participants only via casual employment. While EPs were included in the REABLE-MOC, none were able to be recruited. All participants received physiotherapy, 92% received occupational therapy, and 24% received speech therapy. The AHA delivered interventions to 32% of RAC participants. A total of 1366 AHP hours were delivered within the study, with 1077 hours (79%) dedicated to assessments and interventions. Table 6 provides a summary of the interventions provided during the trial. The modality of interventions were primarily individual sessions (94%) compared to group sessions (5%), with some sessions (1%) provided in an interdisciplinary manner. The remaining 289 hours (21%) were indirect activities and comprised case management meetings (4%), training (4%), travel (2%), and buffer and administrative time (11%).

**Table 6.** Summary of Interventions Provided.

Discipline	Therapist time, h
Physiotherapy	
Individualized exercise programs	340
Resource development	17
Other	9
Subtotal	366
Occupational therapy	
Leisure-based programs	101
Prescription and training of assistive technology	30
Activities of daily living (including upper limb) and functional retraining	172
Staff/care worker training	11
Resource development	24
Research and liaison	22
Subtotal	360
Speech therapy	
Cognitive (language)	27
Voice, speech, and articulation skills	18
Resource development	16
Research and liaison	3
Other	4
Subtotal	68
Allied health assistant	
Individualized exercise programs	29
Total	823

The program was well-received, with participants valuing goal-aligned interventions that provided purpose and empowered re-engagement in meaningful activities, as reported previously. Similarly, AHPs valued delivering the REABLE-MOC, stating that it increased professional satisfaction and growth [*Satisfaction*].

I’ve grown a lot as a professional… because I did not actually imagine that we could do so much more in an aged care setting... So I actually felt proud that I was part of that journey, and that feels great in itself. (PT1)

AHPs noted the REABLE-MOC enabled them to shift their focus from using a generalized approach to providing interventions holistically [*Better practices*], utilizing their full scope-of-practice to produce benefits for the participants (“often the communication of residents isn’t prioritized, so it was really nice to be able to do some of that communication therapy with residents,” ST). Working in this model also shifted AHPs perceptions regarding the benefits they could provide to older people [*Satisfaction*], particularly those living with dementia (“There were some very deteriorating clients in memory support unit. I really didn’t know how they would go, and every one of them has done more than I expected,” OT1). AHPs often reflected on their experience of working in the model as “meaningful and very rewarding” (PT3), resulting from witnessing the positive impacts of goal achievement for the participants [*Better practices; Satisfaction*].

Participant retention was high at 93%, with four participants not completing reassessments due to death (*n* = 1), illness (*n* = 1), or withdrawal (*n* = 2) (see Figure 2). Of the 2144 sessions scheduled, 1804 were attended (84%). Main reasons for non-attendance included participant being unwell (24%), unavailable (16%), isolation or COVID lockdowns (14%), and refusals (14%). A further 19% were cancelled or rescheduled as the AHP was on leave. There were six minor adverse events, five were reports of short-term musculoskeletal pain that led to modification of the exercise program but did not prevent continuation of the program, and one non-injurious fall associated with equipment prescribed.

The multidisciplinary approach, which enabled collaboration in addressing the diverse needs of participants, increased therapy intensity, and supported engagement from the participants, was identified as a crucial feature of the model [*Role of AHP; MDT approach*]. Participants confirmed the importance of the multidisciplinary nature of the model (“think the best aspect of the programme probably is the fact that there’s been three people involved in it, all with different ideas about certain things that I know are going to help me in the long run,” P5, community). Participants also expressed that the frequency of contact with the AHPs was key in providing motivation to engage in the exercises [Role of AHP], with one participating noting, “with this [model], you had two people motivating you twice a week, four times a week, you were motivated” (P1, RAC). Participants received a median time of 16 minutes daily, with median daily costs of $26 (see Table 7 for breakdowns by participant group). Some AHPs reflected on their disappointment with current allied health provision in RAC and community settings, as the contrast in experiences from delivering the best practice–aligned REABLE-MOC highlighted current inadequacies in service provision [Better practices].

I wish this level of therapy was available for everybody in care because I think it is a human rights issue, that people don't have access to what could benefit them. (OT1)

**Table 7.** Allied Health Time and Costs.

	Total	RAC	Community	National average
Median total time	1385 min/23 hrs	1461 min/24.4 hr	942 min/15.7 hr	
Median daily time	16 min	17 min	11 min	4.2 min
Total cost^a^	$2192	$2318	$1492	
Daily cost^a^	$26	$28	$18	$5.41

^a^Prices reported in AUD.

AHPs reported facing challenges that arose from delivering therapy in the RAC setting. These included the RAC staff having limited understanding of the role of different AHPs and that the “concept of retraining is not well recognized here” (OT1), with reports that tasks will continue to be completed on behalf of the resident by care staff unless it is explicitly communicated that residents are working on them [Challenges]. There were also scheduling challenges reported, with difficulties adhering to the intervention timelines among residents’ daily schedules with care and leisure activities, and for community participants, challenges with transport and schedules. AHPs also noted experiencing challenges working with older adults who at times had reduced engagement due to declines in health. They also expressed difficulty with finding the “balance between challenging them [participants] and not challenging them enough” (PT1). Both participants and AHP noted the time-limited nature of the trial as a key barrier to providing long-term benefits to older people. Some participants reported that they would continue to complete the exercise program they had been provided: “Even though we’re at the end of the period, I’m still able to do those exercises and they continue to help” (P5, community). Overall, most expressed notable concern with ending the trial with a lack of maintenance therapy.

Make it longer … all of a sudden, she stopped now… it hasn’t been sustained, unfortunately. (P14, RAC)

The AHP provided feedback to improve the REABLE-MOC, which included increased flexibility with intervention selection such as providing manual therapy, training of RAC staff to support participants’ goals outside of the therapy sessions, and reducing time of sessions to accommodate fatigue experienced by participants.

## Discussion

This study aimed to explore the feasibility, acceptability, and clinical outcomes from delivering the REABLE-MOC for older people in RAC and the community. Clinical outcomes demonstrated increases in physical function, and maintenance in level of frailty and quality of life. Acceptability of the model was driven by personalized goal setting, which produced increased satisfaction and performance with occupations for participants, and increased job satisfaction and growth for staff. Feasibility of delivering the model was demonstrated through delivery of key reablement interventions and economic evaluation, although participants and staff reported concerns regarding long-term sustainability of the REABLE-MOC and outcomes for participants without plans for maintenance support.

Physical function and frailty are key issues impacting independence and meaningful engagement. Considering that people living in residential care become frailer with their increased length of stay (Milte et al., 2022), the high proportion of participants who maintained or reduced level of frailty can be considered a successful outcome. Mobility increased following the REABLE-MOC, although the variability in the sample reduced confidence in this result. The potential improvement in physical function aligns with prior research findings on the effect of reablement programs (Bennett et al., 2022). The substantial focus on physical exercise programs and activities of daily living retraining delivered by physiotherapists and occupational therapists in this study may be associated with its impact on physical function. The program’s ability to maintain the level of frailty and maintain quality of life indicates the potential of reablement approaches to offset further deterioration and optimize well-being in this population.

Personalized goal setting emerged as a crucial component of the REABLE-MOC, as the process enabled meaningful outcomes for both participants and staff. Findings from this study indicate that using COPM to direct interventions supported improvements in ADL function, restored a sense of self-worth in participants, and produced clinically meaningful improvements in both performance and satisfaction sub-scales of COPM. This is an important finding, considering that the transition to RAC is often accompanied with a loss of independence and self-identity (Scott et al., 2024). Providing goal-directed therapy also drove acceptability of the model from staff perspectives, contributing to reports of increased job satisfaction and growth. Considering key factors impacting workforce retention are related to satisfaction and opportunities for growth (Thwaites et al., 2023), the REABLE-MOC may present an opportunity to address workforce retention issues in the sector.

Feasibility of the model was demonstrated. The study implemented key components found in effective reablement interventions, including structured goal setting and complex interventions delivered by a diverse, interdisciplinary team (Buma et al., 2022). The flexible and coordinated approach to the program allowed AHPs to adapt interventions to the health and cognitive changes in the participants, likely contributing to the program’s retention and attendance rates. Economic feasibility emerged as a consideration in this study, given the increased demand for reablement services. In this study, the REABLE-MOC provided a daily median of 17 minutes per RAC participant, closing the gap with the international recommendation of 22 minutes (Eagar et al., 2019), and four times higher than the current daily median provision of 4.16 minutes per resident (Department of Health and Aged Care, 2024c). The limited availability of the speech therapist to deliver the REABLE-MOC impacted the total time available per participant. It is likely that if speech therapy availability was increased, they would be able to deliver similar levels of therapy to participants as physiotherapy and occupational therapists, thereby increasing the daily allied health minutes closer to the recommended 22 minutes. Similarly, the REABLE-MOC in this study increased the daily AH cost to $28 per RAC participant, approximately five times the current national average cost of $5.41 per day per resident (Department of Health and Aged Care, 2024c). Delivering reablement-focused intervention at this level would require substantially more investment than current allied health service expenditures. Efficiency of the REABLE-MOC could be improved through increased utilization of allied health assistants and group-based interventions within the model.

A key limitation was the high number of ineligible residents due to previously accessing allied health services, and recruitment challenges experienced due to COVID-19 outbreaks. The modest sample size and lack of fidelity measurements introduced uncertainty about consistent delivery of interventions. Future studies should include fidelity measures to strengthen findings and explore evaluating the model using controls to compare outcomes for the REABLE-MOC compared to traditional allied health provision. Additionally, the lead author’s prior working relationship with participants may have introduced bias, mitigated through weekly team meetings to ensure objectivity in results.

## Conclusion

Providing a reablement-focused, multidisciplinary allied health model of care aligned with best practice standards can improve physical functioning and maintain quality of life and frailty for older adults. The model was found to be acceptable with high retention and attendance rates as well as positive feedback about the improved outcomes and experiences for both participants and staff. The study was found to be feasible; however, concerns were expressed regarding sustainability of outcomes from the model in the absence of ongoing support. Further evaluation of effectiveness and cost-benefit analysis are necessary to understand the full potential and long-term impact of the model on key outcomes.

## Supplemental Material

Supplemental Material - Multidisciplinary Allied Health Reablement Model of Care for Older People in Residential Aged Care and Community Settings: Mixed-Methods Evaluation Supplemental Material for Multidisciplinary Allied Health Reablement Model of Care for Older People in Residential Aged Care and Community Settings: Mixed-Methods Evaluation by Laura Mo, Katharine Scrivener, Alice Pashley, Diane Gibson, Kasia Bail, Nathan D’Cunha, and Stephen Isbel in Journal of Applied Gerontology.

## Data Availability

The data underlying this study are available in the published article and its online supplementary files.
