# Supplementary material for: Multidisciplinary Allied Health Reablement Model of Care for Older People in Residential Aged Care and Community Settings: Mixed-Methods Evaluation
Source: J Appl Gerontol. 2025 Jun 24;45(4):818–30. doi: 10.1177/07334648251351690 (PMC12989033; doi:10.1177/07334648251351690)
Supplement: Supplemental Material - Multidisciplinary Allied Health Reablement Model of Care for Older People in Residential Aged Care and Community Settings: Mixed-Methods Evaluation [file sj-pdf-1-jag-10.1177_07334648251351690.pdf]

**Interview guide – residents and family members**

Thank you for being involved in this project. The purpose of this interview is to ask you about your opinions and experiences when you were doing the program. I'm hoping to ask you a few questions about that and it should take about 15 minutes.

First of all, can you please tell me how long you have been living here at [name home]

Can you please think back to when you were first referred to the program. You talked to [add health professional] who asked you about what sorts of things you wanted to work on.

In your case I can see [list areas highlighted] were the main things you wanted to look at. My first question relates to that initial process. What did you think of this initial process.

I can see that you have been working with multiple allied health people including [name disciplines].

Can you tell me what you have been doing with [profession] and what you thought of the program.

[repeat the questions above if multiple professions were involved]

Thinking of the overall program now: Do you think the overall program assisted you? Yes/No: In what ways and/or can you give some examples.

What were the best aspects of the program. Can you please give some examples.

What things didn't you like about the program.

Is there anything you suggest changing.

**Interview guide - staff**

Thank you again for agreeing to be interviewed. As you know we have been running an allied health program for residents here at [name home]. This is the first time something like this has been done so we are keen to get the thoughts and opinions of staff here about the program.

First of all, could you please let me know your role here and how long you have been working here.

What do you think about the allied health programs we have been running.

Can you give some examples of where you think it has been beneficial to a resident and why do you think that happened.

Were there any examples of where the program did not work for a resident and why do you think that happened?

What were the best aspects about the program and why.

Would you change anything about the program?

*Table 1. Participant themes, codes, and exemplar quotes*

| Theme | Sub-themes | Exemplar quotes |
|---|---|---|
| Impact and benefits of person-centred interventions | Person-centred nature of the program | "I was asked what I would like to do, and that made me feel a little bit more in control of my life…But being able to say how you'd like to do something makes you feel, again, that you are worthwhile." P7, RAC<br><br>"I feel what we tried to pinpoint is the best for a better life for her is they're the main things really that were already focused on." P8, RAC next of kin<br><br>"Well, I told you what was wrong and you people came up with a way of helping me get it fixed." P9, Community<br><br>"I thought it was very good because it made me think about what was really affecting me and what I needed to improve… I thought the process was very good." P5, Community<br><br>"So that part of it, I think that could be why she liked it, because she felt that she was the boss, she was in control." P10, RAC next of kin<br><br>"Well, I was able to find out where my problems are and then you guys worked on that." P15, RAC |
| | Occupation-based interventions | "The occupational side of it, trying to find one of my hobbies... my favourite hobby is fishing... That's a bit difficult... We just mentioned scrap booking in passing, and now we've done it. I'm thoroughly enjoying it." P1, RAC<br><br>"So looking at the feeding, I think that's really good now and how she's been able to have the right tools to self-feed." P8, RAC next of kin<br><br>"Well I haven't done it [gardening] for ages… When I was living by myself, I did a lot of gardening. I grew all my vegetables, my beans, everything." P3, RAC |
| | Feelings of empowerment and purpose | "It was very enabling." P7, RAC<br><br>"It gave her something to live for. It gave her something to do, even though it was only for a short period… and she was happy with herself. She was proud of herself…She would say, "See, I can do it. I can do it. I can do it" So that was really goodP14, RAC next of kin |

| | | |
|---|---|---|
| | | “Personally, I feel that it worked wonders and I will keep carrying it on, until I can walk down and back, without sitting down. I feel good about it.” P1, RAC<br><br>“That was very clever and it'll give me a purpose to get going again.” P6, Community<br><br>“She gave me exercises to do, which has certainly helped. The beauty of that is that even though we're at the end of the period, I'm still able to do those exercises and they continue to help” P5, Community |
| | Physical, psychological and social benefits | “I'm feeling stronger in the short period of time. I can tell that I'm stronger, so I'm getting around better. Shopping, everything.” P9, Community<br><br>“But then when you go into the group, you find that other people have the same problems as you have, and you can talk together with those people, and together with your therapist find ways of overcoming any problems that you might have.” P7, RAC<br><br>“Well, it helped me to go out for an hour or whatever, and meet other people and do some exercises, which I wouldn't have... always done at home.” P4, Community<br><br>“I think that was me favourite by all of them, because I see different people. See that's another thing. You communicate with other people… I thought that was good.” P3, RAC<br><br>“I really enjoyed the people. They were enjoyable company.” P2, RAC<br><br>“I think the interaction with the other ladies… [they will] have a cup of coffee every week, so I think that interaction was really good.” P11, RAC<br><br>“She had the opportunity to get out of the house.. Also being in a position where she met and spoke to lots of people. The other thing was cognitive skills, that is to follow instructions from the physio, that was fantastic.” P16, RAC NEXT OF KIN<br><br>“My confidence, my mental state, it helped me with that.” P15, RAC<br><br>“So yeah, she seemed much happier.” P10, RAC next of kin |
| | Retained social connects to family, social | “I don't think I'm ever necessarily going get back to being able to play a full game of bowls…that's a lot of walking, but it will enable me at least have roll-ups... I'd happy with that because it's the activity. Usually when you have a roll-up, you're having it with friends anyhow.” P5, Community |

| | | |
|---|---|---|
| | network and community | "I can't lift my mum. She can't walk… now, [the occupational therapist has] shown me that I can remove the wheels. I can easily collapse it, put it in my car, take it out there, and then wheel her in if I have to. So I still can do it." P14, RAC next of kin |
| Diverse role of allied health professionals | AHP's role as a coach and educator | "But with the staff, their patience, and you can see that she's built a bit of a rapport with them. It's comforting to see that she feels safe… So that's been really good." P8, RAC<br><br>"Well, you've got to be motivated. Not having someone to give you a kick in the backside, to get you motivated. With this, you had two people motivating you twice a week. Four times a week you were motivated." P1, RAC<br><br>"And also she's priming me up to make sure I do it a bit more often and it becomes, you know a habit." P6, Community<br><br>"And it showed me that I can do exercises, that I can do movements that I perhaps hadn't thought that I could do before." P7, RAC |
| | Diverse range of skills and attributes were valued by participants | "I find her to be a wonderful leader in that she doesn't push you to do things that are too difficult for you, but she encourages you to have a go at everything." P7, RAC<br><br>"I guess also having someone a bit interested that cared about it all." P6, Community<br><br>"Their attitude of talking to you and just seeing how you felt about this, that and the other." P11, RAC<br><br>"He was great. He was lovely. He was very determined to do things with her and just a lovely person. He would keep me informed all the time with what was going on." P14, RAC<br><br>"But with the staff, their patience, and you can see that she's built a bit of a rapport with them. It's comforting to see that she feels safe… So that's been really good." P8, RAC<br><br>"I think the best aspect of the programme probably is the fact that there's been three people involved in it, all with different ideas about certain things that I know are going to help me in the long run." P5, Community |
| Challenges | Model sustainability | "It wasn't long enough." P5, Community<br><br>"I wish I could continue it longer because I think it's really good for my grandmother to have that support." P8, RAC NEXT OF KIN<br><br>"Make it longer. Use the same people because they're wonderful… then all of a sudden, she stopped now… It hasn't been |

| | | |
|---|---|---|
| | | sustained, unfortunately.” P14, RAC |
| | Limited goal achievement led to dissatisfaction | “So I haven't had any practise…with the walker…my main aim is to do that…my mobility has not improved. It's worsened, I think, and that's upsetting to me.” P2, RAC<br><br>“But I've not achieved any of my goals, I don't think. No, I can't remember what I said exactly, but I know my balance was important, but I haven't achieved that. It's still just the same.” P4, Community |

*Table 2: Allied health professionals' themes, codes, and exemplar quotes*

| Theme | Sub-themes | Exemplar quotes |
|---|---|---|
| Better than usual care and past practices | Goal-driven approach | “I feel like is that it is person-centred. It's not what we think they need. It's what they actually they need. It's their goals. It's their life. It's what do they want to believe, and you're just the facilitator, and you just facilitate that whole process.” PT1<br><br>“We focus on patients goals and it was really specific to them” OT2<br><br>“So addressing different areas of occupation that they're involved in, I do think the program allowed for that.” OT1<br><br>“Yeah, I found that quite enjoyable, actually working with people who wanted to improve and achieve their goals... That was good.” PT2<br><br>“…we were able to identify not only their goals but the main problem areas and they get to prioritise it themselves. So that gives them a bit of some independence to choose what they want to work on and then keeps them motivated PT 3<br><br>“I guess establishing that clear communication at the beginning with, if not the resident, then their families on what to expect from the programme, what we could potentially offer.” PT 4<br><br>“…being able to engage in that task with adapted ways and a different structure, I think just a benefit to him, overall his well-being, taking part in a task was meaningful to him previously that could no longer do and he's doing now” OT2 |
| | Comparison to past practices and usual care | “In the past when I have worked both under AN-ACC [Australian National Aged Care Classification] and ACFI [Aged Care Funding Instrument]..that model doesn't always address participation in society, socialisation with the family and the goals that are most important to the resident.” OT1<br><br>“…primary role here was assessing the residents who would come in the home and asking them what their pain areas |

| | | |
|---|---|---|
| | | were…pretty much a symptomatic relief. And we would not go into details as to what their goals [were]…" PT1<br><br>"…some programmes we run is generalised.." OT2<br><br>"…speech is left to the side unless it's a swallowing concern and often, the communication of residents isn't prioritised. So it was really nice to be able to do some of that communication therapy with residents." SP<br><br>"Oh I wish this level of therapy was available for everybody in care because I think it is a human rights issue, that people don't have access to what could benefit them." OT1<br><br>"So I liked having that flexibility and do what kind of they needed." – PT 2<br><br>"…the goal setting that I've done previously in aged care has usually been focused on whatever we need to address in the care plan. It's very limited, it's very limited…more about transfers and mobility, and equipment." - OT1 |
| | Aspects of program that contributed to improved practice | "…amount of support with resources and being able to acquire based on my plan and say, these are the things that I need for therapy, and having that support, I think that was the best part of the therapy." – OT 2<br><br>"…given all the participants the opportunity of more intensive therapy than they've previously seemed to have had access to." - OT1<br><br>"I feel like I was able to come up with more smart goals, more specific goals with them and it's more relatable to them." PT 3 |
| Importance of multidisciplinary approach | Benefits of multidisciplinary approach for AHPs | "And I think a lot of the physio goals supported my goals as well, whether it's mobility or strength, which they needed to be able to participate in leisure activities as well. So the MDT [multidisciplinary team] structure was really good idea…" OT2<br><br>"I value the fact that physio and OT, and speech, getting together if we've got a complex client or whatever client, and we can hone what areas need to be focused on for the client to achieve their goal." OT1<br><br>"So the communication book, looking at strategies to increase that conversation… look at what other ways do we increase his participation in the home. And then she [occupational therapist] was able to see those benefits as well. So she spoke about how she took him upstairs, used the book. He had a very lovely conversation." SP<br><br>"So then being able to upskill the clinicians in the clinic as well in terms of communication, so that they have a bit more understanding of what they can do to support them was also really nice..." SP |

| | | |
|---|---|---|
| | | "I would regularly and connect with the speech about how OT and speech can deliver well for the clients." OT1 |
| | Benefits of multidisciplinary approach for participants | "I think honestly they just engage more. It's a big part of it. I think they enjoyed having the different people and working on different things kind of at the same time." PT 2<br><br>"you can bounce ideas off of each other during the session so we can get the resident to be more engaged in the session, I'll try one thing, I'll try another thing. We get this person to, despite their cognitive barriers, I think having two people there to help settle some residents, that helped in their engagement." PT 4<br><br>"[The program] made it so much more consistent for this age group... They need the repetition. They need that social interaction consistency as well, so I feel like that helped, too." PT1<br><br>"A little bit of the social engagement for them was good in a way [...]... I think that's maybe a bit overlooked." PT2<br><br>"...the intensity of up to four sessions a week by having two OTs and two physio sessions has motivated people." OT1 |
| Personal and professional satisfaction | Satisfaction working in aged care | "I think it's quite very meaningful because I've been making positive impact to the residents over here and seeing quite a bit of progress and people achieving their goals so it's quite meaningful and very rewarding." PT 3<br><br>"I've grown a lot as a professionally… because I did not actually imagine that we could do so much more in an aged care setting... So I actually felt proud that I was part of that journey, and that feels great in itself." PT1<br><br>"…there were some very deteriorating clients in memory support unit. I really didn't know how they would go, and every one of them has done more than I expected. So, I was pleasantly surprised. And that makes me think we've got a lot of work to do in our memory support area." OT1 |
| | AHPs were able to deliver interventions to their full scope of practice | "I definitely am going to miss being involved in aged care in a way that I haven't been able to do before because it's always just been swallow assessment and feeding training." – SP<br><br>"So, the contribution to the patient. That was what was the most satisfying for me…I was facilitating a meaningful activity and letting them bring their own value to that project. And that was what was so satisfying for me." OT1<br><br>"Yeah, because occupational therapy, there's lots of room for creativity, and I think I always enjoyed the creativity in my personal aspect of myself, so that was really cool for me." OT2 |

| | | |
|---|---|---|
| Challenges in delivering reablement | Challenges the person presented | “…wasn't as engaged in the program due to general health and then obviously cognitive implications as well affected his understanding and engagement.” OT2<br><br>“…a few times where there were unforeseen kind of things happening that impacted a lot [...]…I know he still did okay, but he didn't do really well.” PT2<br><br>“The other challenge, as I said, was with this elderly population, you really want to find a balance between challenging them and not challenging then enough.” PT1<br><br>“So I think the goals that were set, although they may be meaningful for the daughter, they weren't very meaningful or functional for him.” SP<br><br>“I still think she might've misunderstood at the start, because I think she wanted more hands-on therapy for her hip. So I think in a way she didn't feel like it was very useful for her.” PT1 |
| | Challenges the RAC setting presented | “…for care staff, for lifestyle staff, the fact that, they just looked at me strangely, why I was getting involved in their role. So, the role of an OT, educating people about the role of OT…I was starting it at the grassroots level.” OT1<br><br>“That concept of retraining is not well recognised here.” OT1<br><br>“So most of the tasks can be done for them unless they really want to work on something.” OT2<br><br>“Staff weren't always available to come sit down with us… different staff members were present, often hard to communicate those changes across the entire team.” SP<br><br>“Even though you want it to be on a consistent time, sometimes it's not possible….other things, their toileting schedules, can interfere with the treatment as well.” PT1<br><br>“Because sticking to the rigid intervention times was a bit stressful in terms of delivery” OT2 |
| | Improvements to program | “There's an intervention guide and, yeah, I was able to tailor it to a few different situations as well, which was good.” PT2<br><br>“…if there was a little bit more flexibility around what we can and can't do with them and then maybe if I would've probably taken them a little bit slower, paced them properly.” PT1<br><br>“And I feel like if we could include some hands-on component in the programme… we could first address their painful areas” PT1 |

| | | |
|---|---|---|
| | | "So I think in future...whether there's any specific things that we might need to train them on, making sure that the staff that work on that floor or work on a regular basis with that person are well aware and then they can support any new people that come on board." SP<br><br>"…maybe come out twice a week and do 30-minute session… the one hour was either too long in the sense that they would become fatigued quite quickly, or in terms of their own patience, they were fed up with doing the tasks." SP |

Table 2. Checklist for reporting mixed-methods research

| | | |
|---|---|---|
| Rationale and description of MMR design | Provide a clear statement of the study purpose | P3, L7-9 |
| | Explicitly describe the MMR design in accordance with Creswell's (2015) typology and use a diagram to illustrate the relationship and sequence of qualitative and quantitative research components | P3, L13-15 |
| | Justify why the MMR design is appropriate for meeting the study purpose | P3, L15-16 |
| Transparency in describing method details | Describe the study population(s) and samples; (e.g., who, what, how many) | P7, L2-10 |
| | Describe the sampling procedures (including inclusion and exclusion criteria, recruitment) | P4, L17 – P5, L2 |
| | Describe qualitative data collection processes (how often data were collected, who collected the data, what kind of data collection instruments were used, how data were recorded—e.g., notes, transcripts) | P6, L4-10 |
| | Describe quantitative data collection processes (how often data were collected, who collected the data, what kind of data collection instruments were used measurements, validity/reliability) | P5, L4 – P6, L3 |
| | Describe qualitative data analysis processes (coding, single or multiple coders, replication logic, credibility) | P6, L12-18 |
| | Describe quantitative data analysis procedures (missing data and how they are handled, statistical tests used) | P6, L19-25 |
| Integration of qualitative and quantitative research components | Interpret quantitative analysis results in consideration of statistical significance, selection bias, and threats to validity | P7, l14 – P11, L2 |
| | Compare qualitative and quantitative results | P7, l14 – P11, L2 |
| | Address divergencies and inconsistencies between qualitative and quantitative results | P7, l14 – P11, L2 |
